# The Role of Short-Chain Fatty Acids of Gut Microbiota Origin in Hypertension

**DOI:** 10.3389/fmicb.2021.730809

**Published:** 2021-09-28

**Authors:** Yeshun Wu, Hongqing Xu, Xiaoming Tu, Zhenyan Gao

**Affiliations:** Department of Cardiology, The Quzhou Affiliated Hospital of Wenzhou Medical University, Quzhou People’s Hospital, Quzhou, China

**Keywords:** hypertension, blood pressure, short-chain fatty acids, gut microbiota, mechanism, treatment

## Abstract

Hypertension is a significant risk factor for cardiovascular and cerebrovascular diseases, and its development involves multiple mechanisms. Gut microbiota has been reported to be closely linked to hypertension. Short-chain fatty acids (SCFAs)—the metabolites of gut microbiota—participate in hypertension development through various pathways, including specific receptors, immune system, autonomic nervous system, metabolic regulation and gene transcription. This article reviews the possible mechanisms of SCFAs in regulating blood pressure and the prospects of SCFAs as a target to prevent and treat hypertension.

## Introduction

Hypertension is a global public health problem as well as an important risk factor for cardiovascular and cerebrovascular diseases (Rossier et al., 2017; Mills et al., 2020). Increase in the ageing population and lifestyle risk factors consequently increases the global prevalence of hypertension. As of 2010, 1.38 billion people (31.1% of the global adult population) suffer from hypertension (Mills et al., 2016). Consistent with the trend of hypertension prevalence, the number of cardiovascular and cerebrovascular deaths increased significantly from 1990 to 2015; hypertension is responsible for 40.1 and 40.4% of deaths caused by heart disease deaths and stroke deaths, respectively (Forouzanfar et al., 2017; Furie, 2020; Mills et al., 2020). Hypertension is a complex and multifactorial disease affected by both genetic and environmental factors (Rossier et al., 2017; Mills et al., 2020). In the genome-wide association study of Ehret et al. involving 342,415 individuals, numerous single gene variants related to blood pressure control and hypertension were identified, but these specific causal genes only explained a small proportion of the systolic pressure variation between individuals (<5%; Ehret et al., 2016). Therefore, identification of new diagnosis and treatment targets based on hypertension pathogenesis is urgently needed.

Gut microbiota and its metabolites play an important role in human health and diseases by affecting the body’s metabolism, immunity, nervous system and endocrine homeostasis (Fenneman et al., 2020; Verhaar et al., 2020; Yoo et al., 2020; Wu et al., 2021). Hypertension occurrence is often accompanied with gut microbiota imbalance, including decreased diversity, altered enterotype distribution and variation in bacterial populations (Li et al., 2017). It is mainly characterised by the increase in *Klebsiella*, *Prevotella*, *Coprobacillus* and *Enterobacter* populations and the decrease in *Anaerotruncus*, *Coprococcus*, *Ruminococcus*, *Clostridium*, *Roseburia*, *Blautia* and *Bifidobacterium* populations, which reduce ratio of *Firmicutes/Bacteroidetes* and the production of short-chain fatty acid (SCFA; Li et al., 2017; Verhaar et al., 2020). These observations provide a new perspective for hypertension diagnosis and treatment. SCFAs, which are the main metabolites produced by intestinal bacteria, ferment dietary fibre in the gastrointestinal tract. SCFAs can be effectively absorbed by the intestinal mucosa; they act as a source of energy, a regulator of gene expression, a participant in cell metabolism and a signal molecule recognised by specific receptors, thereby having an important impact on blood pressure regulation (Jin et al., 2019; Poll et al., 2020; Naqvi et al., 2021). This article summarises the possible mechanisms of SCFAs in regulating blood pressure and reviews the prospect of SCFAs as a target for hypertension prevention and treatment.

## SCFAs and Blood Pressure

Short-chain fatty acids are an important link between the host and gut microbiota, which comprise different bacteria in the intestine, especially anaerobic bacteria, through dietary fibre fermentation (Kumar et al., 2020; Ge et al., 2021). SCFAs are organic fatty acids with fewer than six carbon atoms, and acetate, propionate and butyrate are considered the most important and biologically effective ones, accounting for 95% of the SCFAs produced by the gut microbiota (He et al., 2020). SCFAs are mainly produced in the colon and cecum, with a total concentration of approximately 150mmol/l. As the most abundant anion in the colon, SCFAs are absorbed in a concentration-dependent manner, transported to the portal vein by various transporters and then migrated to other organs through blood circulation (Nicholson et al., 2012; Liu et al., 2021). The proportion of the three main SCFAs (acetate, propionate and butyrate) is roughly 3:1:1, and they differ in their sources, distribution and potential effects on host physiology (He et al., 2020; Kumar et al., 2020; Liu et al., 2021). Acetate is the main SCFA in the colon produced by most of the *Enterococcus* species and is easily absorbed and transported to the liver. Propionate, produced by *Bacteroidetes*, *Acidaminococcus* and *Salmonella*, is also absorbed and transported to the liver, promoting intrahepatic gluconeogenesis. Furthermore, butyrate, which is produced by *Clostridium*, *Eubacterium* and *Roseburia*, can be used as the energy source of the intestinal mucosa and regulates cell proliferation and differentiation, with the hydroxylation product β-hydroxybutyrate (BHB) as its main effective component in the circulation (Wong et al., 2006; Nicholson et al., 2012; Louis and Flint, 2017; Sasaki et al., 2020; Liu et al., 2021).

Despite their low peripheral circulation concentration (0.1–10mM), as signal molecules, SCFAs are involved in different physiological and pathological processes of the host (Natarajan and Pluznick, 2014). Several animal model studies revealed that SCFAs can regulate blood pressure. In spontaneously hypertensive rats (SHR) and deoxycorticosterone acetate salt induced hypertensive rats (DHR), both high-fibre diet and acetate and propionate supplementation can significantly reduce the blood pressure levels (Pluznick et al., 2013; Marques et al., 2017). Yang et al. analysed the intestinal bacterial genome of stool samples and observed that the SCFA-producing microbiota was significantly less abundant in SHR than in the normal controls (Yang et al., 2015). Similarly, Holmes et al. found that SCFAs significantly correlated with blood pressure levels in East Asian and western population samples (Holmes et al., 2008). In a case report of patients with refractory hypertension, minocycline administration to inhibit intestinal microbiota could produce a powerful anti-hypertensive effect (Qi et al., 2015), which may be closely related to *Firmicutes/Bacteroidetes* reduction by minocycline (Pluznick, 2013; Yang et al., 2015). SCFAs not only directly affect hypertension progression but also regulate several hypertension-related syndromes, such as obesity, insulin sensitivity and diabetes (de la Cuesta-Zuluaga et al., 2018; Mandaliya and Seshadri, 2019; Zhi et al., 2019). Acetate and butyrate can be used as substrates for lipid synthesis, whereas propionate can serve as the medium of liver gluconeogenesis (Liu et al., 2021). Compared with the normal-weight group, the obesity group had an altered SCFA composition in the faeces in which the proportion of acetate was relatively lower (Schwiertz et al., 2010). SCFAs are increasingly proven to be widely involved in body-weight regulation, energy metabolism balance, lipid metabolism and other pathophysiological processes (den Besten et al., 2015; Hu et al., 2018; Barrea et al., 2019), which cross-correlated with hypertension.

## Possible Mechanism of SCFAs Regulating Blood Pressure

Short-chain fatty acids have an important impact on blood pressure regulation, whereas hypertension occurrence is typically accompanied with the decrease in SCFAs production. Moreover, a number of potential mechanisms have been proposed to explain this association, including specific receptors, immune system, autonomic nervous system, metabolic regulation, cell senescence and gene transcription.

### G-Protein-Coupled Receptors

Through receptor binding, SCFAs can directly regulate blood pressure. The currently discovered SCFA receptors are mainly G-protein-coupled receptors (GPR), such as GPR41, GPR43, GPR109A and olfactory receptor (Olfr) 78 (Priyadarshini et al., 2018; Muralitharan et al., 2020; Poll et al., 2020; Yao et al., 2020). Interestingly, SCFAs binding to different receptors play a diametrically opposite role in blood pressure regulation (Bolognini et al., 2016; Pluznick, 2017).

GPR41 and GPR43 are widely distributed throughout the body. They are activated when they bind to acetate, propionate and butyrate (Kim et al., 2013; Kimura et al., 2020; Muralitharan and Marques, 2021), whereas GPR109A specifically binds to butyrate and BHB (Thangaraju et al., 2009). GPR41 could be expressed in vascular smooth muscle cells and endothelial cells; its expression is necessary for SCFA-mediated vasodilation (Natarajan et al., 2016). Natarajan et al. added propionate to the diet of GPR41^−/−^ and GPR41^+/−^ mice; consequently, propionate produced an anti-hypertensive effect in the latter and an opposite effect in the former (Natarajan et al., 2016). Blood pressure was significantly higher in GPR41-knockout mice than in wild-type mice, with a more pronounced systolic pressure level (Natarajan et al., 2016). After GPR41 knockout, mice exhibited thickening of aorta and increase in vascular collagen, consequently causing vascular fibrosis and hypertension (Natarajan et al., 2016). Similarly, Onyszkiewicz et al. demonstrated that butyrate can pass into the bloodstream through the gut–vascular barrier and act on GPR41/GPR43 to relax the mesenteric artery, thereby significantly mitigating hypertension (Onyszkiewicz et al., 2019).

The Olfr is a seven-pass transmembrane GPR (known as Olfr78 in mice and OR51E2 in humans) that also serves as a receptor for SCFAs, especially acetate and propionate (Segers et al., 2019; Kotlo et al., 2020). In mice, Olfr78 is mainly distributed in the kidneys and blood vessels; when activated, it can increase blood pressure, possibly because it affects the vascular smooth muscle cells in renal afferent arterioles and peripheral blood vessels (Pluznick et al., 2013; Miyamoto et al., 2016). The renal afferent arteriole is the main site for renin secretion and storage. In Olfr78-knockout mice, plasma renin and blood pressure levels are decreased (Pluznick, 2014; Miyamoto et al., 2016). Treating Olfr78-knockout and non-knockout mice with an identical dose of propionate, the former showed lower blood pressure levels; this may be caused by the stimulation of cyclic adenosine monophosphate (cAMP) production in glomerular cells, resulting in renin release (Pluznick et al., 2013; Pluznick, 2014). In peripheral vascular smooth muscle cells, Olfr78 expression may affect the baseline blood pressure (Hsu et al., 2018). Moreover, propionate mediated by Olfr78 receptors can produce a blood pressure-boosting effect, mainly by resisting its powerful anti-hypertensive effect caused by other receptors or pathways (Pluznick et al., 2013; Pluznick, 2017).

**Figure 1:**
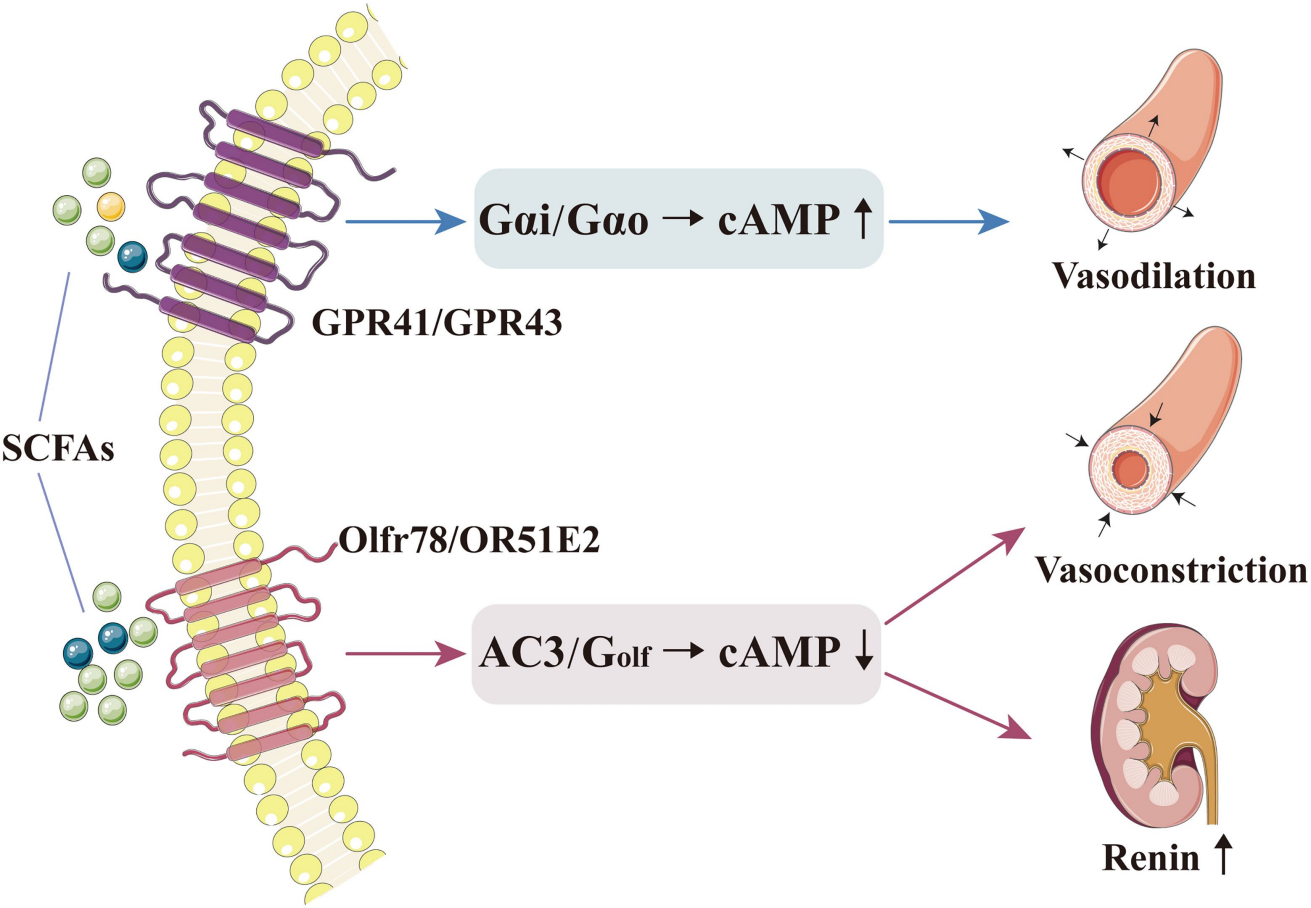
SCFAs can directly regulate blood pressure by binding to their receptors. Basal concentration of SCFAs could activate GPR41/GPR43, which triggers Gαi and/or Gαo to decrease cAMP, thereby inducing vasodilation and lowering the blood pressure level. A higher concentration of SCFAs could activate Olfr78, which triggers AC3 and Golf in the olfactory signalling pathway to induce cAMP production, thereby increasing renin release and inducing vasoconstriction. AC3, adenylate cyclase type 3; cAMP, cyclic adenosine monophosphate; GPR, G-protein-coupled receptor; Olfr, olfactory receptor; and SCFA, short-chain fatty acid.

Olfr78 and GPR41 produce effects through different G protein-α subunits and second-messenger systems; Olfr78 activates adenylate cyclase type 3 (AC3) and G_olf_ in the olfactory signalling pathway to induce cAMP production, while GPR41 and GPR43 activate Gαi and/or Gαo to decrease cAMP (Pluznick et al., 2013). After being activated by SCFAs, these receptors are coupled with different second messengers and generate opposite effects on blood pressure (Pluznick et al., 2013). Therefore, the mechanisms and physiological effects of SCFAs on blood pressure regulation are complex and diverse, and the abovementioned contrasting effect may explain the blood pressure fluctuations mediated by SCFA level alteration (Figure 1). Pluznick et al. reported that exogenous propionate injection (0.1mmol) into mice could cause a large (20mm Hg) and rapid (within 1–2min) blood pressure drop, which would then return to the normal level within 5min; after the injection, the blood pressure level was lower in Olfr78^−/−^ mice than in wild-type mice (11.9±1.6mm Hg *vs*. 5.5±0.5mm Hg; *p*<0.000013). In addition, to verify the role of GPR41 receptor in blood pressure regulation, they used the same method and found that after exogenous propionate injection (10mmol, maximum physiological dose), the blood pressure level slightly decreased in GPR41^+/−^ mice (2.9±1.6mmHg) and moderately increased in GPR41^−/−^ mice (4.5±2.4mmHg; Pluznick et al., 2013; Pluznick, 2014, 2017). Thus, after exogenous SCFA administration, the blood pressure of Olfr78^−/−^ mice would be greatly reduced. The reason could be that SCFAs could only bind to GPR41 to exert an anti-hypertensive effect, with no Olfr78 antagonism; in contrast, SCFAs in GPR41^−/−^ mice could only bind to Olfr78, with no GPR41, thereby increasing blood pressure (Pluznick et al., 2013; Pluznick, 2014, 2017). The opposite effect of SCFAs on blood pressure regulation may be related to the different sensitivity of Olfr78 and GPR41 towards SCFAs. Plasma SCFAs at basal concentrations (0.1–0.9mmol) could activate GPR41 to induce vasodilation and lower blood pressure; conversely, SCFAs with a higher concentration (0.9mmol) could activate Olfr78 to increase renin release and blood pressure levels (Miyamoto et al., 2016). Given that Olfr78 can adjust the inappropriate GPR41/GPR43-mediated hypotension resulting from excessively elevated circulating SCFA levels, the acute hypotensive effect of propionate is accentuated at low physiological doses (Pluznick, 2014). Hence, the role of different SCFA concentrations in different tissues must be determined.

### Immunoregulation

The immune system and excessive inflammation have been increasingly proven to participate in hypertension development. T-lymphocyte subsets, such as T helper (Th) 1, Th2, Th17, regulatory T (Treg) and CD8+ T cells, are involved in regulating blood pressure and tissue damage (Wenzel et al., 2016; Ren and Crowley, 2019). SCFAs are important regulators of immune pathways, including intestinal and immune homeostasis, inflammatory cell biology and inflammatory response (Li et al., 2018b; Parada Venegas et al., 2019; Ratajczak et al., 2019). Bartolomaeus et al. observed that in mice with hypertension induced by angiotensin (Ang) II, propionate attenuated the response of various T cells to Ang II, such as Th17 and memory T-cell reduction, thereby lowering the blood pressure level; this process was confirmed to be Treg-dependent (Bartolomaeus et al., 2019). Moreover, butyrate can regulate hypertension occurrence and development through the immune response. *In vitro*, butyrate can decrease the interleukin (IL)-6 and tumour necrosis factor-α (TNF-α) levels caused by Ang II and induce Treg differentiation *in vivo* and *in vitro*; this SCFA can also reverse the elevated Th17 and IL-17 levels in patients with hypertension (Furusawa et al., 2013; Ohira et al., 2013; Singh et al., 2014; Wang et al., 2017; Kim et al., 2018). In mice, a high-fibre diet or acetate supplementation can significantly reduce systolic and diastolic pressure and improve cardiac fibrosis and left-ventricular hypertrophy, related to the downregulation of the signal transduction of the proinflammatory cytokine IL-1 in the kidney (Marques et al., 2017). For salt-sensitive hypertension, the exogenous supplementation of BHB precursor inhibits the formation of the renal inflammasome NOD-, LRR- and pyrin domain-containing protein 3, thereby attenuating hypertension (Chakraborty et al., 2018).

The anti-inflammatory effects of SCFAs (especially butyrate) may be mediated by histone deacetylase (HDAC) inhibition in vascular endothelial cells (Chang et al., 2014; Li et al., 2018a; Yang et al., 2020). HDAC inhibition contributes to the prevention of vascular inflammation and related diseases; in SHR, HDAC activation is closely related to hypertension (Cardinale et al., 2010; Chun, 2020; Li et al., 2020). Verdin et al. proved that as an HDAC inhibitor, BHB could increase the histone acetylation level, promote the expression of the antioxidants factor forkhead box O3a (FOXO3a) and metallothionein 2 (MT2) and ultimately protect the body from oxidative stress (Shimazu et al., 2013). Furthermore, the butyrate and valerate levels in the faeces of patients with preeclampsia were significantly decreased; correspondingly, butyrate directly downregulated lipopolysaccharide-induced hypertension in preeclampsia rats by regulating macrophage function and inhibiting HDAC (Chang et al., 2014). In addition, butyrate injection in *Npr1^+/−^* mice significantly lowered the blood pressure levels and reduced renal inflammation and fibrosis by inhibiting HDAC (Kumar et al., 2017).

**Figure 2:**
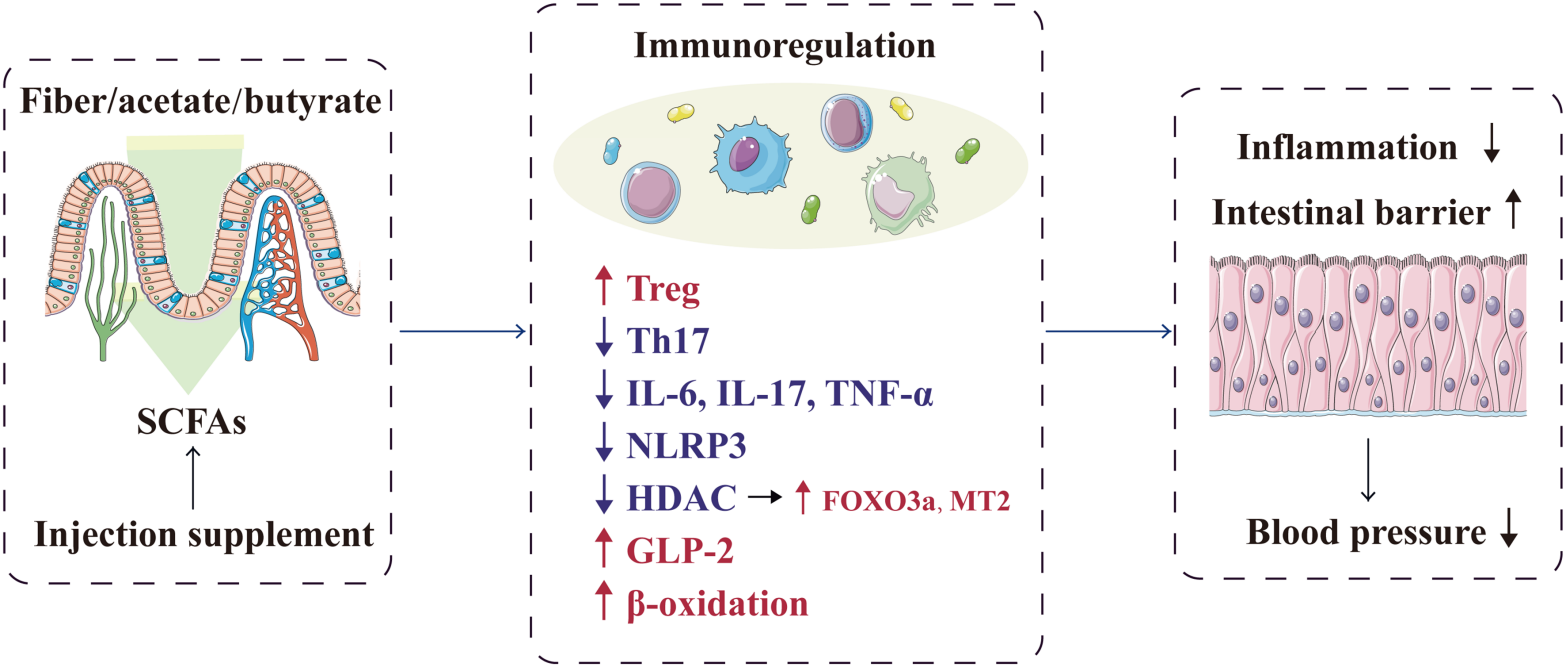
SCFAs play a local protective role in blood pressure regulation by regulating the intestinal barrier and immune response. FOXO3a, forkhead box O-3; GLP-2, glucagon-like peptide 2; HDAC, histone deacetylase; IL, interleukin; MT2, metallothionein-2; NLRP3, NOD-, LRR- and pyrin domain-containing protein 3; SCFA, short-chain fatty acid, TH17, T helper cell 17; TNF-α, tumour necrosis factor-α; and Treg, regulatory T cell.

In addition to providing energy to the intestinal epithelium, SCFAs promote the integrity of the intestinal epithelium and help repair the damaged epithelium (D’Souza et al., 2017). Thus, inhibition of these mechanisms are inhibited would lead to uncontrolled infiltration between the lumen and adjacent vessels, thereby inducing systemic inflammation and subsequently participating in hypertension pathogenesis (Jama et al., 2019). SCFAs can also promote the secretion of anti-inflammatory intestinal hormones, including glucagon-like peptide 2 (GLP-2; Onal et al., 2019). As a specific intestinal growth factor, GLP-2 can promote the growth of normal intestinal mucosa, repair the damaged intestinal epithelium and protect the intestinal mucosal barrier (Brubaker, 2018; Chang et al., 2021). Moreover, SCFAs, especially butyrate, can enhance the β-oxidation process of the cells in the intestinal mucosa, consume oxygen in the intestinal lumen and then create a favourable intestinal microenvironment to promote the growth of beneficial bacteria and inhibit the proliferation of potential pathogenic microorganisms; ultimately, the immune inflammatory response related to hypertension is alleviated, and target organ damage is relieved (Cani, 2017; Guan et al., 2020). Similarly, Kim et al. confirmed that butyrate treatment in mice with Ang II-induced hypertension can ameliorate microbial imbalance and intestinal barrier dysfunction, thereby reducing the mean arterial pressure (Kim et al., 2018). Therefore, the metabolites of gut microbiota can protect host’s health locally by regulating the intestinal barrier and immune response (Figure 2).

### Autonomic Nervous System

**Figure 3:**
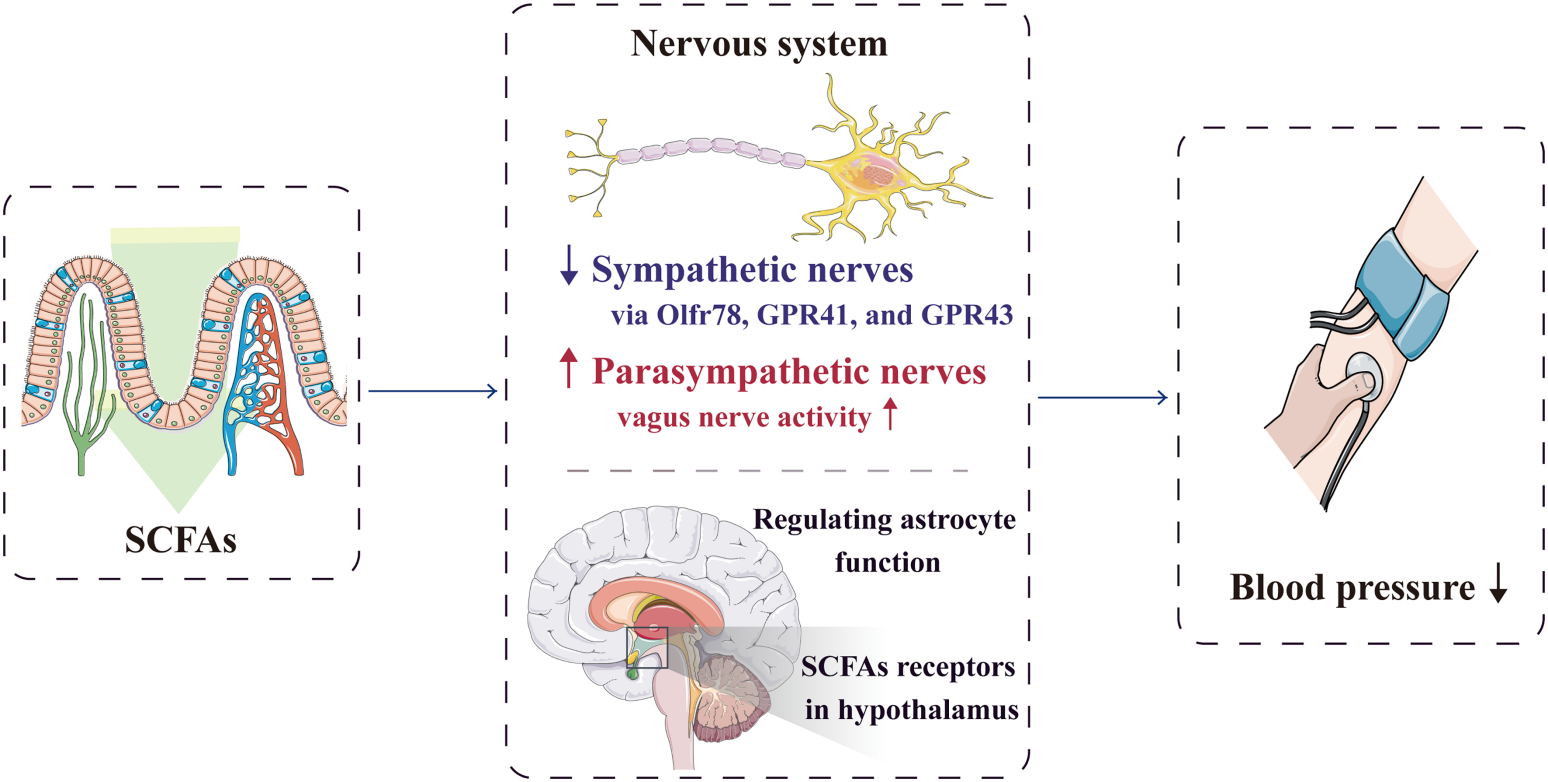
SCFAs can regulate blood pressure through the nervous system. By acting on the receptors expressed in the sympathetic ganglia, SCFAs can directly regulate the sympathetic nervous system. They can also significantly activate vagal afferent neurons, which facilitate SCFAs to regulate blood pressure. Furthermore, SCFAs can directly act on the central nervous system to reduce the blood pressure. GPR, G-protein-coupled receptors; Olfr, olfactory receptor; and SCFA, short-chain fatty acid.

Hypertension occurrence is closely related to autonomic nervous dysfunction (Mancia and Grassi, 2014; Kalla et al., 2016). In SHR, long-term stimulation of cardiac vagus nerve preganglionic neurons could lower blood pressure (Moreira et al., 2018), whereas short-term vagus nerve stimulation could improve the prognosis of rats with salt-sensitive hypertension (Annoni et al., 2019). The microbiota–gut–brain axis is a complex neuro–humoral communication network that maintains body homeostasis; it consists of gut microbiota, enteric nervous system, central nervous system and autonomic nervous system and its related sympathetic and parasympathetic branches. Through this axis, SCFAs can regulate blood pressure (Dalile et al., 2019; Zubcevic et al., 2019; Figure 3). The sympathetic and parasympathetic ganglia express SCFA receptors, such as Olfr78, GPR41 and GPR43. By acting on the receptors expressed in the sympathetic ganglia, SCFAs can directly regulate the sympathetic nervous system (Kimura et al., 2011; Nohr et al., 2015), and through the receptors expressed in the parasympathetic ganglia, SCFAs can affect the neural feedback in the gut (Zubcevic et al., 2019), Therefore, SCFAs participate in the neural regulation mechanism of blood pressure.

Lal et al. reported that SCFAs, especially butyrate, can directly activate the afferent nerve fibres of vagus nerve after being absorbed into the nerve endings of intestinal mucosal lamina propria, which help SCFAs in blood pressure regulation (Lal et al., 2001). Moreover, Goswami et al. showed that SCFAs can significantly activate vagal afferent neurons by increasing phosphorylation, with the butyrate>propionate>acetate effect (Goswami et al., 2018). After transection of the sub-phrenic vagus nerve and pre-treatment of the colon with a nonspecific antagonist of GPR41/GPR43, the anti-hypertensive effect of butyrate is weakened; therefore, the stimulation of butyrate on the vagal afferent fibres may partly cause the anti-hypertensive effect (Onyszkiewicz et al., 2019).

Additionally, butyrate can cross the blood–brain barrier through specific transporters and directly act on the central nervous system to regulate blood pressure; for instance, butyrate improves the function of astrocytes in the central nucleus (Vijay and Morris, 2014; Yang et al., 2018). SCFA receptors are also present in the para-ventricular nucleus, and injecting butyrate into the lateral ventricle can significantly reduce blood pressure levels in both the SHR and control groups (Yang et al., 2019). In addition, butyrate receptors in the hypothalamus of SHR are less expressed, resulting in decreased reactivity; therefore, the role of butyrate in blood pressure regulation is affected (Yang et al., 2019).

### Metabolism

**Figure 4:**
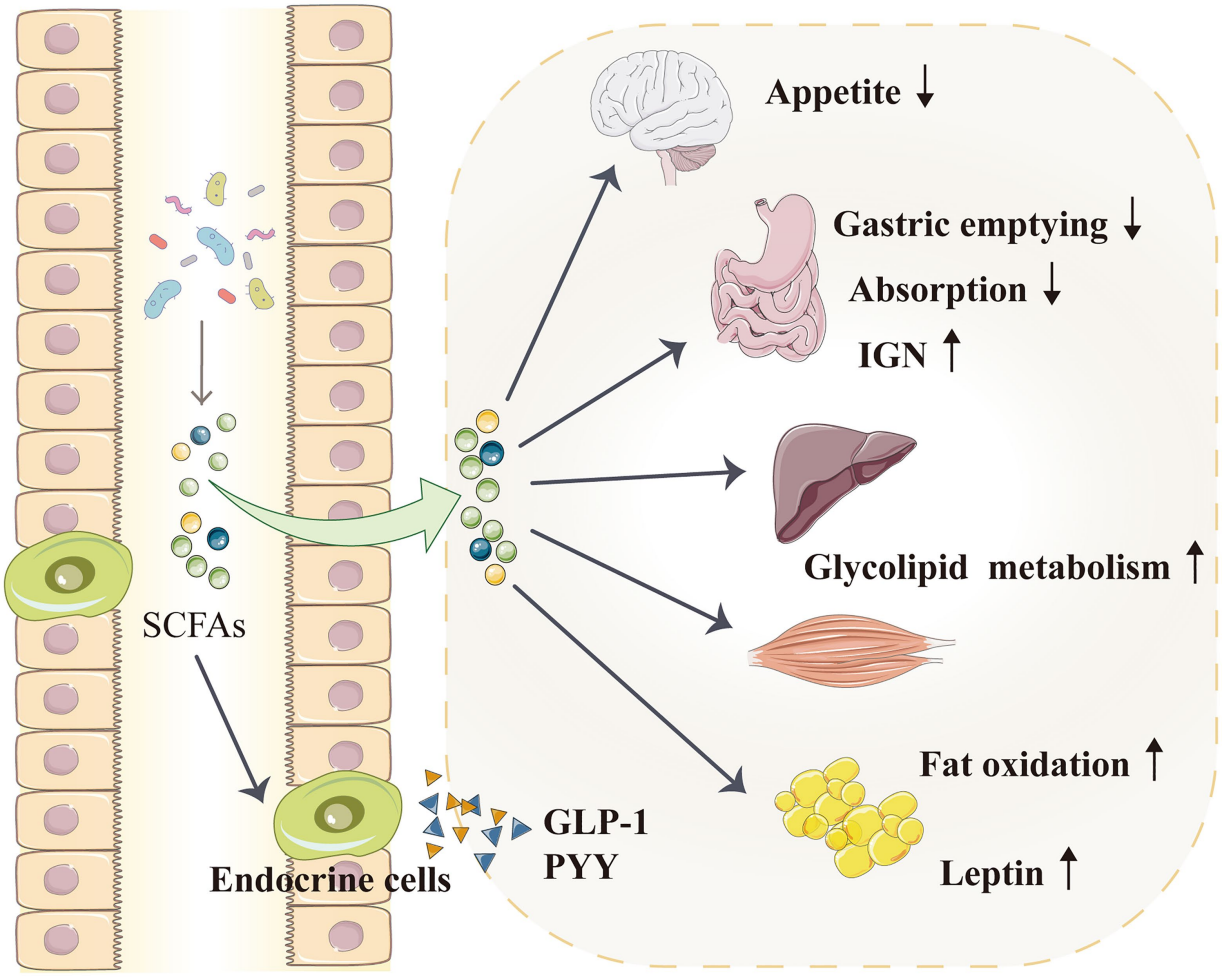
SCFAs indirectly regulate blood pressure by affecting the metabolism. SCFAs can activate IGN, which is involved in maintaining normal blood glucose levels and energy homeostasis. SCFAs enter the liver and muscles to improve glycolipid metabolism. SCFAs can also directly enter the blood–brain barrier and act on the hypothalamus to inhibit appetite. Furthermore, SCFAs promote the secretion of intestinal hormones, such as GLP-1 and PYY, which can slow down gastric emptying and reduce food energy absorption. SCFAs can also increase fat oxidation and promote the secretion of leptin from adipocytes; leptin is a typical metabolic hormone that reduces food intake, increases energy release and reduces body mass. GLP-1, glucagon-like peptide 1; IGN, intestinal gluconeogenesis; PYY, peptide tyrosine tyrosine; and SCFA, short-chain fatty acid.

According to epidemiological and animal data analyses, hypertension and other metabolic disorders, such as diabetes and obesity, have an extremely close and reciprocal causal relationship (Velarde and Berk, 2005; Katsimardou et al., 2020; Litwin and Kulaga, 2021). The role of SCFAs in obesity and metabolic regulation (glucose and lipid metabolism) has attracted increasing attention, and these metabolites may indirectly regulate blood pressure by participating in metabolism (Figure 4; Frampton et al., 2020; He et al., 2020; Machate et al., 2020). After being produced by gut microbiota, SCFAs are initially used by intestinal epithelial cells as an energy source, and they can activate intestinal gluconeogenesis (IGN), which is important in maintaining normal blood glucose level and energy homeostasis (De Vadder et al., 2014). SCFAs also circulate through the blood vessels and enter the liver and muscles to regulate energy metabolism. The SCFA propionate is a good precursor for glycolipid and protein syntheses (Wilson et al., 2017). Acetate, which is also an SCFA, is a matrix for cholesterol synthesis (den Besten et al., 2013); it can directly cross the blood–brain barrier and act on the hypothalamus to inhibit appetite (Frost et al., 2014; Hartstra et al., 2015). Moreover, oral (rather than intravenous) butyrate can reduce food intake and improve glucose and lipid distribution through the gut–brain axis in animals (Li et al., 2018c), whereas oral propionate can increase fat oxidation in humans (Chambers et al., 2018).

Through the GPRs on the surface of intestinal endocrine cells, SCFAs promote the secretion of intestinal hormones, such as glucagon-like peptide-1 (GLP-1) and peptide tyrosine tyrosine (PYY; Zhang et al., 2019a; Ren et al., 2020; Wang et al., 2020; Nishida et al., 2021). In particular, GLP-1 enhances glucose tolerance and regulate metabolism (Suzuki and Aoe, 2021), whereas PYY increases satiety, reduces food intake, regulates intestinal movement and slows down gastric emptying to improve body metabolism (Freire and Alvarez-Leite, 2020; Nishida et al., 2021). GPRs affect the influence of gut microbiota on the body’s energy, i.e. when intestinal GPR activation is inhibited, food energy absorption would be reduced (Samuel et al., 2008). In overweight people, propionate promotes PYY and GLP-1 secretion, improves insulin sensitivity and reduces food intake; its long-term use can control weight gain and reduce abdominal fat (Chambers et al., 2015b).

Short-chain fatty acids can also promote leptin secretion from adipocytes; leptin is a typical metabolic hormone that reduces food intake, increases energy release and reduces body mass (Chambers et al., 2015a; Wang et al., 2020). Insulin signalling in adipocytes can also be inhibited by SCFAs, leading to the prevention of insulin-mediated fat accumulation and promotion of the metabolism of unbound lipids and glucose in other tissues (Kimura et al., 2013; Jiao et al., 2020). A pig model study suggested that oral SCFAs—acetic, propionic and butyric acids—can decrease serum triglyceride, total cholesterol and low-density lipoprotein cholesterol levels and increase serum GLP-1, PYY and leptin levels, thereby reducing fat deposition (Jiao et al., 2018, 2020). Evidence from mouse and human research also supports that SCFAs regulate lipogenesis and attenuate lipolysis (Ohira et al., 2016; Ivan et al., 2017; Aguilar et al., 2018).

### Cell Senescence

**Figure 5:**
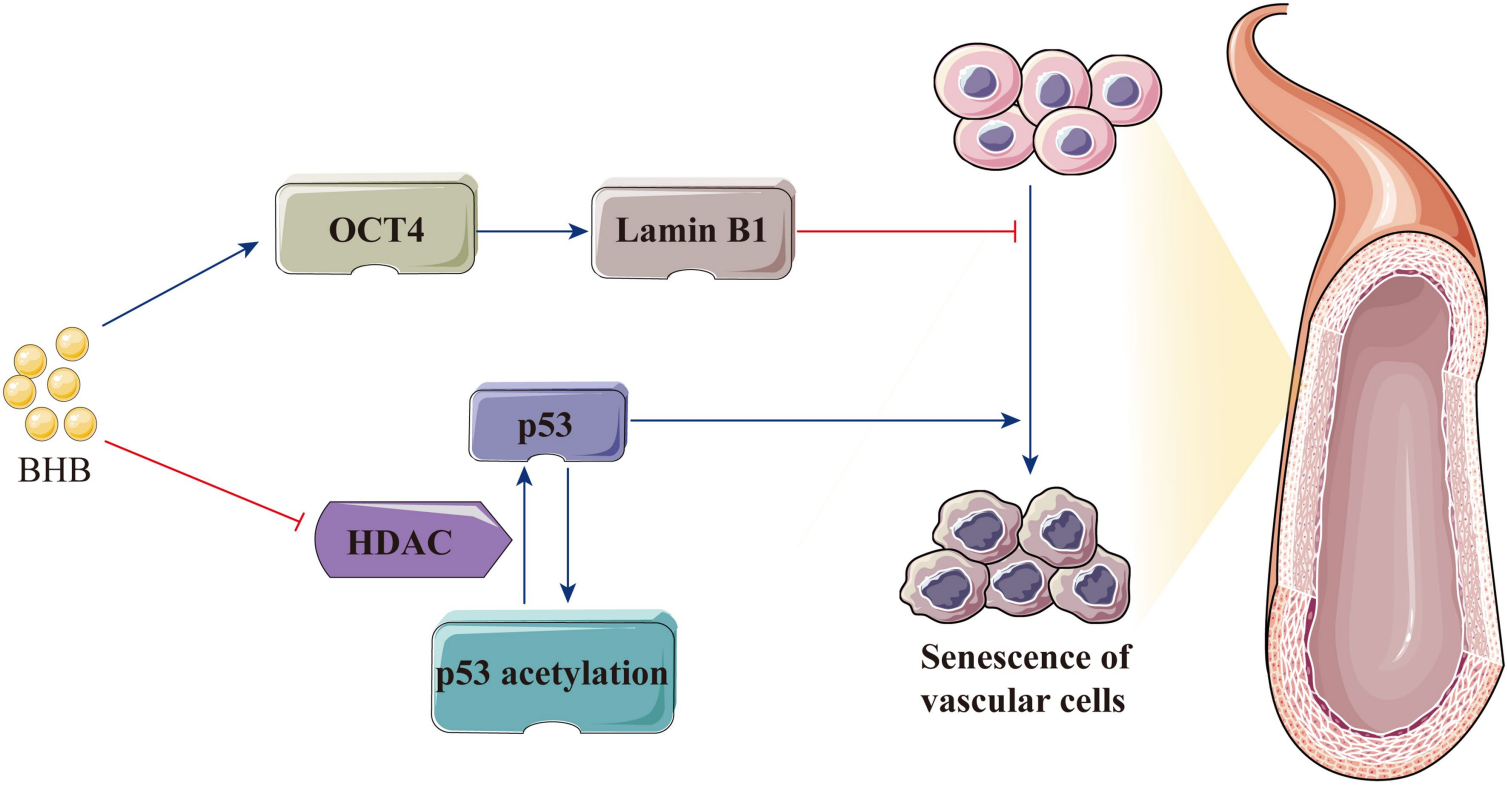
BHB protects the body against hypertension by delaying vascular stiffness associated with endothelial cell senescence. BHB indirectly increases the lamin B1 level by enhancing the expression of OCT4 and increases p53 acetylation by HDAC inhibition, thereby attenuating cellular apoptosis and delaying endothelial cell senescence. BHB, β-hydroxybutyrate; HDAC, histone deacetylase; and OCT4, octamer-binding transcriptional factor 4.

Decreased vascular elasticity and compliance lead to increased vascular wall stiffness, which is the most important feature of hypertension; furthermore, hypertension occurs when endothelial cell senescence leads to the increase in intimal stiffness (Kaess et al., 2012; Tian and Li, 2014; Li et al., 2019). In cardiovascular diseases, P53 is a key regulator of the senescence of endothelial cells and vascular smooth muscle cells (Men et al., 2021). As a vascular protective factor, BHB increases p53 acetylation *via* HDAC inhibition and weakens its activity; consequently, the expression of the downstream genes *p21* and *PUMA* decreases to attenuate cellular apoptosis (Newman and Verdin, 2014; Liu et al., 2019). BHB also significantly inhibits stress-induced premature ageing and replicative senescence through the p53-independent pathway. Furthermore, BHB indirectly increases lamin B1 level by enhancing the expression of the transcription factor octamer-binding transcriptional factor (OCT) 4, which is important for preventing senescence induced by DNA damage (Han et al., 2018; Mendelsohn and Larrick, 2018). Therefore, the protective effect of BHB on hypertension is possibly mediated by delaying vascular stiffness associated with endothelial cell senescence (Figure 5).

### Gene Transcription

**Figure 6:**
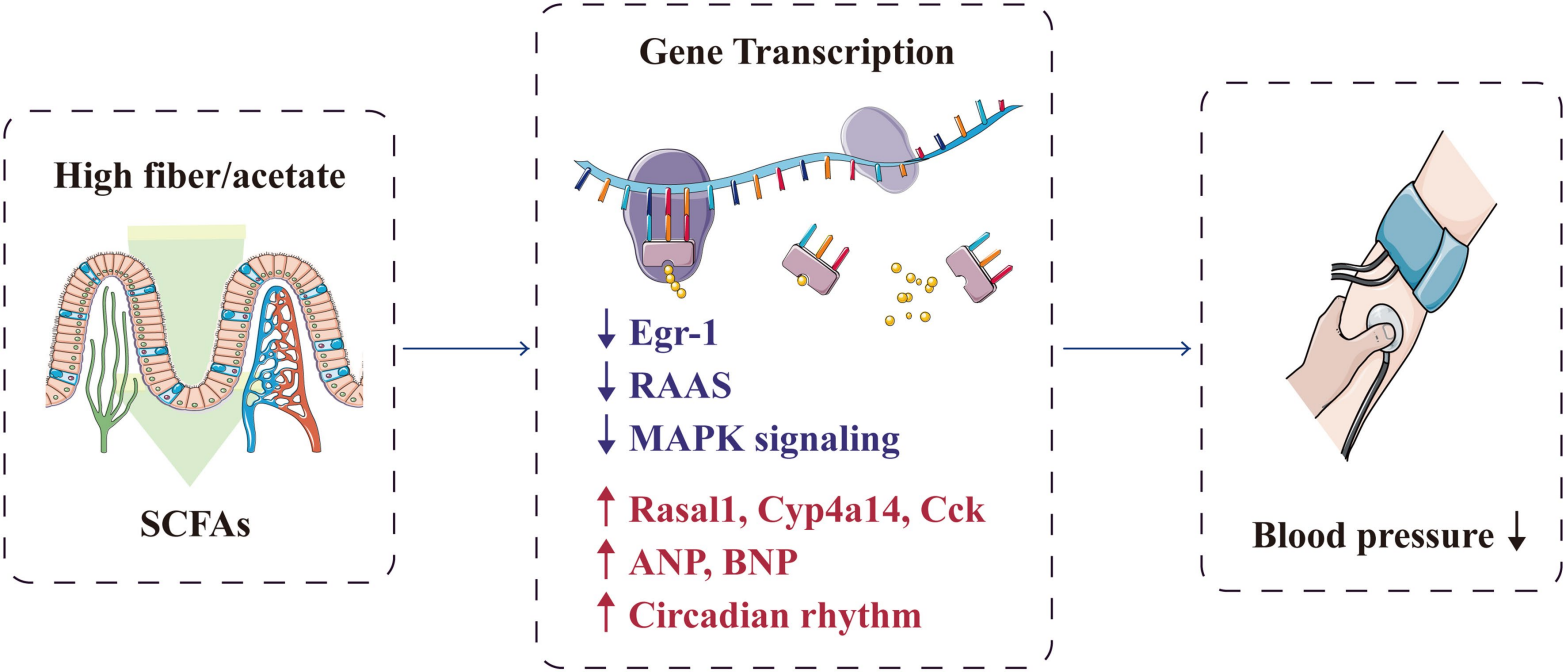
SCFAs protectively regulate the transcription of genes associated with hypertension. ANP, atrial natriuretic peptide; BNP, brain natriuretic peptide; Egr-1, early growth response-1; MAPK, mitogen-activated protein kinase; RAAS, renin–angiotensin–aldosterone system; and SCFA, short-chain fatty acid.

Short-chain fatty acids can prevent hypertension by regulating the hypertension-associated gene transcription in the gut–heart–renal axis (Figure 6). Marques et al. conducted RNA sequencing on the heart and kidney transcriptomes of mice fed with a standard fibre diet, a high-fibre diet or an acetate diet for 3weeks. They found that high-fibre and acetate intake affected the expression of genes associated with heart disease and hypertension, including Rasal1 [associated with renal fibrosis (Bechtel et al., 2010)], Cyp4a14 [encoding a protein that regulates body fluid absorption through sodium channels (Nakagawa et al., 2006)] and Cck [associated with the anti-inflammatory process (Miyamoto et al., 2012)], and genes that regulate the renin–angiotensin–aldosterone system (Marques et al., 2017). SCFAs also upregulated genes related to circadian rhythm and downregulated genes related to the mitogen-activated protein kinase signalling pathway (Marques et al., 2017; Segers et al., 2019). Cardiovascular pathology is mainly regulated by the gene encoding early growth response-1 (Wang et al., 2014; Ho et al., 2016), which is significantly downregulated in the kidney and heart with fibre intake or acetate supplementation (Marques et al., 2017). Furthermore, SCFAs can promote the expression of genes encoding proteins involved in blood pressure regulation; these genes include atrial natriuretic peptide and brain natriuretic peptide (Thorburn et al., 2015).

## SCFAs as a Target for the Prevention and Treatment of Hypertension

**Figure 7:**
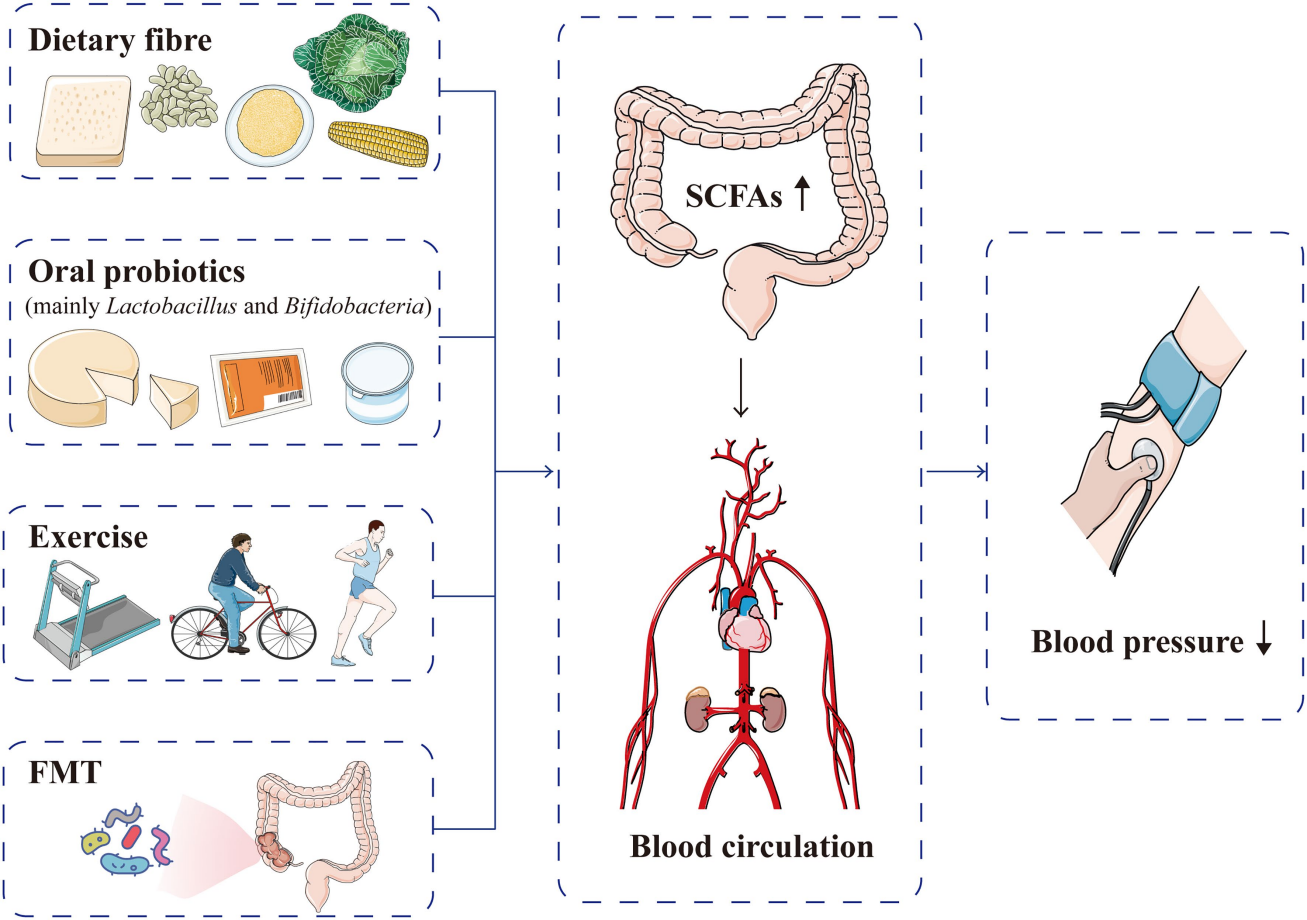
Current methods used to intervene and regulate SCFA production for blood pressure normalisation. Dietary fibre, oral probiotics, exercise and FMT can increase the production of SCFAs, which are absorbed into the blood circulation and subsequently lower blood pressure levels. FMT, faecal microflora transplantation; SCFA, short-chain fatty acid.

Short-chain fatty acids are closely related to hypertension occurrence and prognosis, and increasing the production of SCFAs can improve cardiovascular homeostasis, suggesting a potential target for reducing cardiovascular risk (Jadoon et al., 2018; Almeida et al., 2021; Figure 7). Although the available data are still very preliminary, SCFAs have been found to be related to the metabolism of anti-hypertensive drugs, and regulation of the gut microbiota metabolites, such as SCFAs, may help attenuate drug resistance in patients with refractory hypertension (Qi et al., 2015; Zhernakova et al., 2016). Internal and external factors, such as gut microbiota, diet and intracellular regulatory factors, dynamically regulate SCFAs and the corresponding acylation (Chen et al., 2020). The current main methods for intervening and regulating SCFA production are as follows:

### Dietary Adjustment

The effect of diet on blood pressure has been investigated in recent decades. A high-salt diet alters the production of SCFAs by disrupting the composition of gut microbiota; thus, hypertension develops (Bier et al., 2018). Meanwhile, a high-fibre diet and dietary fibre supplementation could increase the circulating levels of SCFAs and subsequently reduce blood pressure (Myint et al., 2018; Zhai et al., 2018; Zhao et al., 2018). In an experiment using a hypertensive rat model, Marques et al. indicated that the systolic and diastolic pressure levels were significantly lower in DHR with high-fibre diet or acetate supplementation than in the control group (Marques et al., 2017). A high-fibre diet can reduce systolic pressure from 116±19mmHg (baseline) to 91±5mmHg and diastolic pressure from 75±5mmHg to 58±5mmHg, whereas acetate supplementation can reduce systolic pressure from 116±19mmHg to 85±9mmHg and diastolic pressure from 75±5mmHg to 54±5mmHg (Marques et al., 2017). Hence, a high-fibre diet can change the composition of gut microbiota by increasing the level of *Bacteroidetes* and acetate-producing enterobacteria, which exert a hypotensive effect through acetate production (Marques et al., 2017). Similarly, in clinical studies, dietary supplementation with SCFAs has also achieved beneficial effects. Streppel et al. systematically reviewed 24 clinical trials (published from 1966 to 2003) investigating on blood pressure reduction by fibre diet. They found that an average daily intake of 11.5g of fibre food could reduce systolic pressure by 1.13mm Hg (95% CI, −2.49 to 0.23) and diastolic pressure by 1.26mm Hg (95% CI, −2.04 to −0.48); these results were more significantly observed in people aged over 40years and those with hypertension (Streppel et al., 2005). Although the specific effects and mechanisms of dietary structure on SCFAs and hypertension remain obscure, increasing the intake of dietary fibre generally increases the proportion of SCFA-producing bacteria in the intestinal microbes and relatively augments SCFA production, thereby reducing the risk of hypertension.

### Oral Probiotics

Probiotics are living microorganisms that are beneficial to the health of the host; clinically, they mainly consist of *Lactobacillus* and *Bifidobacteria* (Wegh et al., 2019; Markowiak-Kopec and Slizewska, 2020). When carbohydrate is lacking, *Bifidobacteria* can produce acetate and formate through glycolysis; when carbohydrate is sufficient, acetate and lactate are produced (Aoki et al., 2017). In rat models, *Lactobacillus* intake could lower blood pressure levels, related to the improvement of the intestinal barrier function and the production of peptides to inhibit angiotensin-converting enzyme (Nakamura et al., 1995; Robles-Vera et al., 2020). In a recent meta-analysis of 23 randomised controlled studies, probiotics could lower systolic pressure levels by 3.05mmHg and diastolic pressure levels by 1.51mmHg in 2037 adults with or without hypertension (Qi et al., 2020). However, the anti-hypertensive effect of probiotics is inconsistent in different populations; this effect is more evident in Japanese patients and patients with hypertension, suggesting a relationship with genetic or environmental factors (Dong et al., 2013; Qi et al., 2020). The anti-hypertensive effect of probiotics can only last for a short period of time (8 or 10weeks), with only a slight reduction in blood pressure (Qi et al., 2020); hence, probiotic supplementation may be used as an adjuvant therapy. Currently, the regulation of SCFA production by probiotics for hypertension treatment has not yet been investigated. Thus, future studies should focus on clarifying how probiotics influence blood pressure by gut microbiota metabolites.

### Exercise

Exercise is an effective and safe nondrug treatment for many metabolic diseases. Moderate exercise combined with diet control can improve cardiovascular disorders, such as hypertension (Keating et al., 2020; Saco-Ledo et al., 2020). Through exercise, the composition of gut microbiota and the metabolites changes (Monda et al., 2017; Mohr et al., 2020). Allen et al. demonstrated that compared with the non-exercise group, the SCFAs in stool samples increased significantly after 6weeks of endurance exercise in lean participants, but not in those with obesity; when the exercise training was stopped, the exercise-induced changes of microbiota were basically reversed (Allen et al., 2018). The effect of moderate exercise on SCFAs provides a new possible mechanism to explain how exercise prevents and treats hypertension, suggesting an interesting research topic.

### Faecal Microflora Transplantation

Faecal microflora transplantation (FMT) has been applied in the clinical treatment of inflammatory bowel disease and metabolic syndrome; it increases the diversity of gut microbiota, thereby changing the composition of metabolites (Weingarden and Vaughn, 2017; Costello et al., 2019; Zhang et al., 2019b). Animal model studies have explored the effect of FMT on hypertension. Li et al. transplanted stool samples separately from healthy and hypertensive individuals into sterile mice and reported that the systolic and diastolic pressure levels of mice receiving faecal transplants from patients with hypertension were higher than those of mice receiving faecal transplants from healthy individuals; hence, blood pressure increase could be transmitted through gut microbiota (Li et al., 2017). Although relevant clinical reports on hypertension treatment with FMT remain unavailable, recent studies have shown that patients with FMT have a significantly increased production of SCFAs (Seekatz et al., 2018). With further clarification of the relationship between SCFAs and blood pressure, FMT could be a new treatment for hypertension.

## Conclusion

As dietary habits and the living environment change, the prevalence of hypertension increases. Gut microbiota has been gaining considerable attention. SCFAs, which are gut microbiota metabolites, can be involved in hypertension occurrence and development through various pathways, such as specific receptors, the immune system and the autonomic nervous system. However, most of the studies on SCFAs in hypertension are phenotypic descriptions, and their specific molecular mechanisms remain unclear. Considering the differences in intestinal and body functions between rodents and humans, the research findings, which are mostly from rodents, cannot always be extrapolated to humans. Additional studies are needed to clarify the potential effects and internal mechanisms of SCFAs on hypertension, which could provide basic information for the novel prevention and treatment methods of hypertension.

## Author Contributions

XT and ZG: conceptualisation. YW: writing—original draft preparation. YW, HX, XT, and ZG: writing—review and editing. ZG: supervision. All authors have read and agreed to the published version of the manuscript.

## Funding

This work was funded by Clinical Research Fund Project of Zhejiang Medical Association (2021ZYC-A46) to XMT; Guiding Scientific and Technological Project of Quzhou (2021007) to ZG.

## Conflict of Interest

The authors declare that the research was conducted in the absence of any commercial or financial relationships that could be construed as a potential conflict of interest.

## Publisher’s Note

All claims expressed in this article are solely those of the authors and do not necessarily represent those of their affiliated organizations, or those of the publisher, the editors and the reviewers. Any product that may be evaluated in this article, or claim that may be made by its manufacturer, is not guaranteed or endorsed by the publisher.
